# Elucidating the Molecular Mechanisms of Physiological Fruit Abscission in *Actinidia arguta* Through Comparative Transcriptomics and Transient Genetic Transformation

**DOI:** 10.3390/plants14111645

**Published:** 2025-05-28

**Authors:** Pengqiang Yuan, Yanli Wang, Yining Sun, Guoliang Liu, Hongyan Qin, Shutian Fan, Yiping Yan, Bowei Sun, Wenpeng Lu

**Affiliations:** Institute of Special Animal and Plant Sciences, Chinese Academy of Agricultural Sciences, Changchun 130112, China; 82101222242@caas.cn (P.Y.); wangyanli@caas.cn (Y.W.); 82101225210@caas.cn (Y.S.); 82101232245@caas.cn (G.L.); qinhongyan@caas.cn (H.Q.); fanshutian@caas.cn (S.F.); 82101225211@caas.cn (Y.Y.); sunbowei1020@163.com (B.S.)

**Keywords:** *Actinidia arguta*, comparative transcriptomics, fruit abscission, transient transformation

## Abstract

*Actinidia arguta* (*A. arguta*) is valued for its nutritional richness, but physiological fruit abscission severely limits production efficiency in elite cultivars. To unravel the molecular basis of this process, we compared two cultivars: abscission-prone ‘KL’ and abscission-resistant ‘JL’. During fruit development, ‘KL’ exhibited an earlier decline in auxin (AUX) levels within the fruit abscission zone (FAZ), coupled with persistently higher ethylene (ETH) concentrations and polygalacturonase (PG) activity compared to ‘JL’. Comparative transcriptomics identified abscission-related genes enriched in plant hormone signaling (AUX, ETH, ABA, JA, BR), starch/sucrose metabolism, and photosynthesis pathways. AUX signaling diverged predominantly during early development, while ETH, BR, and JA pathways varied across multiple stages. Exogenous applications of plant growth regulators (ethephon, 2,4-D, methyl jasmonate, and 2,4-epibrassinolide) and transient overexpression of key genes (*AaETR1*, *AaERF035*, *AaPME68*, *AaPP2C27*, *AaMYC1*, and *AaPMEI10*) validated their roles in modulating hormone crosstalk and cell wall remodeling. Overexpression of *AaERF035* and *AaPME68* likely accelerated abscission by enhancing ETH biosynthesis and pectin degradation, while *AaPMEI10* and *AaMYC1* potentially delayed abscission via suppression of cell wall-modifying enzymes. This study elucidates the hormonal and transcriptional networks governing fruit abscission in *A. arguta*, providing insights for targeted breeding and cultivation strategies to mitigate yield loss.

## 1. Introduction

Physiological abscission, defined as the programmed process by which plant tissues or entire organs are systematically separated from the parent plant, is a critical adaptive mechanism integral to plant development and survival strategies [1]. The phenotypic expression of orderly plant growth that we observe is fundamentally shaped by the intricate regulatory network governing both cellular expansion and abscission events. Plant abscission occurs at a specific site termed the abscission zone (AZ), which is typically established during organ formation [2]. Plant abscission is a precisely regulated process that occurs in four stages. This ensures the orderly separation of plant organs while protecting the parent plant from potential damage. Initially, the formation and differentiation of the AZ occur; here, cells specialize into compact, tightly arranged entities during organogenesis, in parallel with the development of organs arising from the shoot apical meristem (SAM) [3]. Upon receiving specific signals related to abscission, AZ cells enter the second stage by initiating the detachment sequence. The third stage is characterized by heightened activity of cell wall-modifying enzymes, leading to the degradation of the pectin-rich middle lamella, which diminishes intercellular adhesion and promotes cell separation [4]. The process culminates in the fourth stage with the formation of a protective layer at the site of abscission and dedifferentiation of adjacent tissues, allowing the plant to maintain structural integrity and prepare for subsequent growth following organ loss [5].

During the second stage of abscission, plant hormones act as primary effectors, regulating AZ activation through a complex signaling network that is based on their synthesis, catabolism, and transport [6]. The balance between ethylene (ETH) and auxin (AUX) levels is a decisive factor in the process of organ abscission [7]. The AUX concentration gradient at the proximal and distal ends of AZ cells dictates the initiation of abscission. Elevated AUX levels reduce the sensitivity of AZ cells to ETH [8]. Abscisic acid (ABA) functions as a sensor for nutrient deficiency stress, regulating AUX and ETH levels by inhibiting AUX transport and modulating ACC (1-aminocyclopropane-1-carboxylic acid) levels to enhance ETH production. Elevated ABA concentrations thereby promote fruit abscission in many species, including citrus [9,10], lychee [11], cherry [12], and apple [13]. During the aging process, increasing levels of jasmonic acid (JA) influence abscission by inhibiting cell wall polysaccharide synthesis in AZ cells and enhancing the activity of cell wall-modifying enzymes within the pedicel AZ [14]. Brassinolide (BR) has been demonstrated to induce a delay in abscission by specifically binding to the promoters of ACS (1-aminocyclopropane-1-carboxylate synthase) and ACO (1-aminocyclopropane-1-carboxylate oxidase), thereby inhibiting their transcriptional activity and reducing ETH biosynthesis [15].

*Actinidia arguta*, commonly known as the mini kiwi or hardy kiwi, is a dioecious plant species indigenous to China, with a natural distribution primarily across the northeastern, northwestern, and northern regions [16]. It is also found in Russia, Japan, Korea, the United States, New Zealand, and other regions. Renowned for its delightful sweetness and nutrient-dense composition, *A. arguta* has earned the title of a superfruit and is widely cherished by consumers [17]. The *A. arguta* variety ‘Kuilv’ (‘KL’), developed by the Institute of Special Animal and Plant Sciences under the Chinese Academy of Agricultural Sciences, exhibits robust growth with a fruit set rate exceeding 95% [18]. This cultivar is particularly notable for its commercial attributes, including the production of large fruit (weighing up to 32 g each) and a high vitamin C content (up to 430 mg per 100 g of fruit) [19]. However, despite these favorable commercial qualities, ‘KL’ faces significant challenges in production due to fruits detaching easily upon maturation (drop rate reaching up to 70% during ripening). This complicates harvest management and poses considerable obstacles to the broader promotion of this variety. Another *A. arguta* variety, ‘JiaLv’ (‘JL’), exhibits similar fruit morphology and development periods to ‘KL’ but has inferior commercial quality [20]. Preliminary data from our team indicate that nearly half of ‘JL’ fruits remain on the tree three days after reaching maturity, demonstrating a pronounced resistance to abscission.

To investigate the abscission characteristics and regulatory mechanisms in *A. arguta* and to inform future scientific and targeted breeding efforts, we selected ‘KL’ and ‘JL’ due to their markedly different abscission behaviors. By monitoring physiological changes during fruit development and conducting comparative transcriptomics between these two varieties, this study aims to identify key plant hormone signaling pathways and differential genes associated with fruit abscission. The study will elucidate the physiological and molecular mechanisms of fruit drop and explore the roles of various plant hormones in this process, providing a theoretical basis for the effective prevention of fruit abscission.

## 2. Materials and Methods

### 2.1. Plant Materials

In this study, fifteen-year-old plants of *A. arguta*, including the easy-abscission cultivar ‘KL’ and the hard-abscission cultivar ‘JL’, were cultivated at the *Actinidia arguta* Resource Nursery of the Institute of Special Animal and Plant Sciences, Chinese Academy of Agricultural Sciences, located in Zuojia Town, Jilin City, Jilin Province. The selected *A. arguta* plants were maintained under uniform cultivation practices and integrated pest management conditions, ensuring consistent growth environments and comparable agroclimatic conditions.

From July to September 2023, a systematic evaluation of the developmental progression of *A. arguta* was conducted. Sampling occurred on eight occasions between 45 and 88 days post-anthesis (DPA). Samples were collected from both fruits and fruit abscission zones (FAZs). For FAZ sampling, a blade sterilized with 75% ethanol was used to excise a 3–5 mm^2^ square region centered on the pedicel scar at the fruit apex, retaining 1–3 mm of the pedicel. The AZ tissue was carefully dissected from the top downward to ensure complete removal of the abscission zone, yielding a sample containing residual pedicel tissue, intact AZ, and minimal adjacent fruit flesh.

In the second year, *A. arguta* plants used for transient transformation and application of plant growth regulators were maintained in the same location and under identical management conditions. Three representative plants, chosen for their uniform size and vigor, served as the experimental cohort. Each sampling event included three independent biological replicates to ensure statistical reliability. Upon collection, all samples were immediately flash-frozen in liquid nitrogen and stored at −80 °C to preserve tissue integrity for subsequent analyses.

### 2.2. Scanning Electron Microscopy (SEM) Analysis

After isolating fresh FAZ specimens, the samples were rinsed gently with PBS (pH 7.4) and immediately fixed in electron microscopy-grade fixative at room temperature for 2 h. The fixed samples were stored at 4 °C. The tissue blocks were washed three times with 0.1 M PB (pH 7.4), 15 min each. The samples were treated with 1% OsO4 in 0.1 M PB (pH 7.4) for 1–2 h at room temperature, followed by repeated PB washes. Dehydration was performed through a graded ethanol series (30% to 100%, 15 min per concentration) before incubation with isoamyl acetate. The samples were subjected to critical-point drying, mounted on metal stubs using carbon adhesive, and sputter-coated with a thin gold layer (10–15 nm). Imaging was conducted using scanning electron microscopy under high vacuum conditions with secondary electron detection.

### 2.3. Quantification of Plant Hormones and Activity Assays of Cell Wall-Modifying Enzymes

FAZ samples (1 g) were ground in 9 mL of 0.01 mol/L PBS (pH = 7.4) in an ice bath. The homogenate was centrifuged at 4 °C and 6000 rpm for 10 min. The supernatant was collected and stored in a refrigerator until use. The concentrations of plant hormones and the activities of cell wall-modifying enzymes were detected with the Plant Indole-3-Acetic Acid (IAA) ELISA Kit, the Plant Ethylene (ETH) ELISA Kit, the Plant Abscisic Acid (ABA) ELISA Kit, the Plant Cellulase (CEL) ELISA Kit, the Plant Polygalacturonase (PG) ELISA Kit, and the Plant Pectin Methylesterase (PME) ELISA Kit (Shanghai Enzyme-linked Biotechnology Co., Ltd., Shanghai, China). Each sample was performed in three replications, and the OD value was measured by a spectrophotometer at a wavelength of 450 nm.

### 2.4. RNA-Seq and Data Analysis and Weighted Gene Co-Expression Network Analysis (WGCNA)

The RNA extraction for transcriptomic analysis included FAZ samples collected at 45 DPA, 64 DPA, 72 DPA, 76 DPA, 84 DPA, and 92 DPA. These samples were sequentially designated as A period (45 DPA), B period (64 DPA), C period (72 DPA), D period (76 DPA), E period (84 DPA), and F period (92 DPA) for further analysis, with each period encompassing both ‘KL’ and ‘JL’ cultivars. A total of 36 libraries, representing the 12 FAZ samples with three biological replicates each, were constructed for RNA-seq. The purity, concentration, and integrity of the RNA samples were assessed to ensure the quality of the transcriptomic sequencing samples. The sequencing libraries were sequenced on the Illumina sequencing platform by Genedenovo Biotechnology Co., Ltd. (Guangzhou, China). Based on the alignment results from HISAT2, transcripts were reconstructed using StringTie, and the expression levels of all genes in each sample were quantified using RSEM. DEGs were identified using a threshold of |fold change| ≥ 2 and false discovery rate (FDR) < 0.05. For WGCNA, transcripts per kilobase per million mapped reads (tpm) values were utilized as input data, with low-expression genes (tpm ≤ 5) excluded to enhance reliability. RNA-seq and WGCNA analyses were conducted through Omicsmart (http://www.omicsmart.com, accessed on 16 March 2025), a cloud-based bioinformatics platform enabling real-time interactive data processing and visualization.

### 2.5. Quantitative PCR (qPCR)

FAZ samples (0.1 g) were rapidly frozen in liquid nitrogen, and total RNA was extracted using the FastPure Plant Total RNA Isolation Kit (RC401-01, Vazyme, Nanjing, China). cDNA was synthesized from the total RNA using the HyperScript^TM^ First-Strand cDNA Synthesis Kit (K1072, Apexbio Technology LLC, Shanghai, China). qPCR analysis was performed using the HotStart^TM^ Universal 2X FAST Green qPCR Master Mix (K1172, Apexbio Technology LLC, Shanghai, China), with *Actin* (*Achn107181*) as the internal reference gene. Each experiment included three biological replicates for each FAZ sample, and the relative mRNA expression levels were calculated using the comparative Ct (ΔΔCt) method. The primer design lists for the genes are provided in Tables S2 and S3.

### 2.6. Treatment with Different Plant Growth Regulators

Fifteen-year-old *A. arguta* trees of consistent growth, which included two varieties, were selected. Different plant growth regulators were applied to the trees: 2000 mg/L ethephon (ETH), 100 mg/L 2,4-D, 0.1 mmol/L methyl jasmonate (MeJA), and 0.1 mg/L 2,4-epibrassinolide (EBR), on 1 August 2024 (50 DPA). The control group was sprayed with water. On each tree treated with plant growth regulators, three branches were randomly marked. FAZ samples were collected for SEM sectioning 7 days post-treatment. Thirty days post-treatment, FAZ samples were collected for physiological measurements and qPCR analysis, using *Actin* (*Achn107181*) as the reference gene.

### 2.7. Overexpression of Candidate Genes

The full-length CDSs of *AaAt4g24780*, *AaPMEI10*, *AaPP2C27*, *AaETR1*, *AabglX*, *AaMYC1*, *AaERF035*, and *AaPME68* were amplified from the cDNA library of *A. arguta*. The pCambia1302 expression vector (HedgehogBio, Shanghai, China) was selected, with BamHI as the restriction enzyme site. The EHA105 strains harboring the constructs 35S:*AaAt4g24780*, 35S:*AaPMEI10*, 35S:*AaPP2C27*, 35S:*AaETR1*, 35S:*AabglX*, 35S:*AaMYC1*, 35S:*AaERF035*, 35S:*AaPME68*, and the empty vector were infiltrated into the *A. arguta* cultivar ‘JL’, and physiological changes in the FAZ were monitored. In the transient expression experiments, qPCR was performed to monitor the expression levels of overexpressed genes in FAZ, with *Actin* (*Achn107181*) being used as the reference gene. Each experiment included three biological replicates and three technical replicates. The primers used in this study are listed in Table S3.

## 3. Results

### 3.1. Physiological Responses of the FAZ During Development in Two A. arguta Varieties

**Figure 1:**
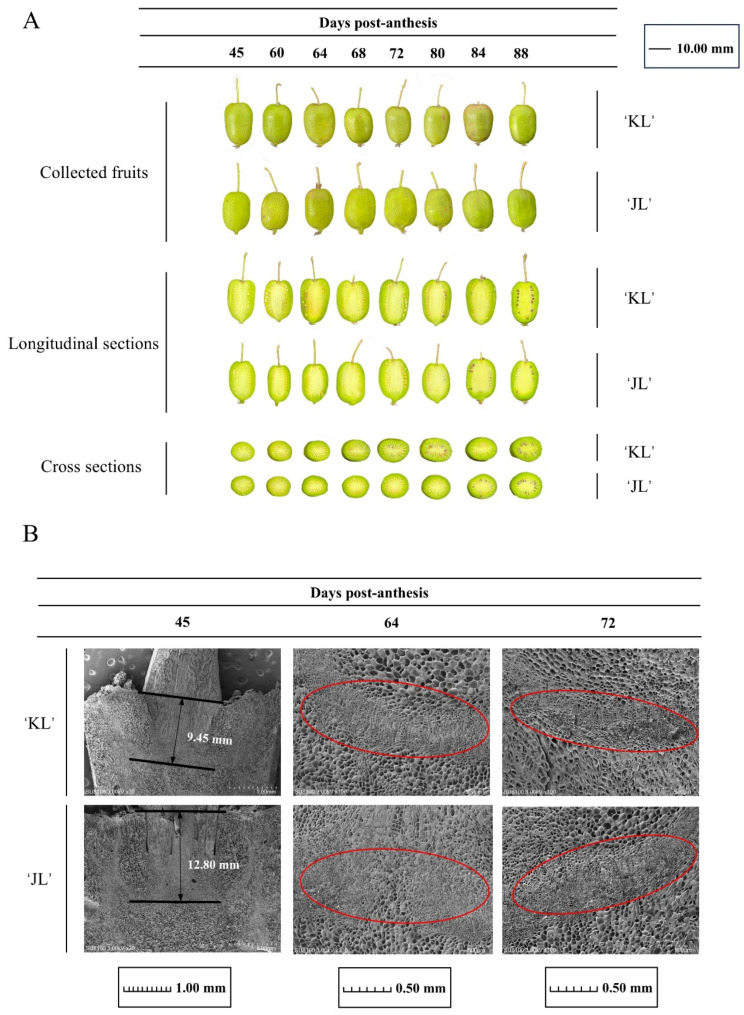
Developmental characterization and AZ morphology in *A. arguta* fruits. (**A**) Comparative analysis of two cultivars (labeled on the right) across eight developmental stages (45–88 DPA). Top panel: Whole fruits exhibit parallel phenotypic progression, validating their suitability as comparable experimental materials. Middle and bottom panels: Longitudinal and cross-sectional views document synchronized seed maturation in both cultivars. Labels are provided in the upper right corner. (**B**) Scanning electron microscopy (SEM) of AZ morphology in both cultivars at three stages (45, 64, 72 DPA). Scale bars: 1 mm for 45 DPA, 0.50 mm for 64 and 72 DPA. Images reveal cultivar-specific cellular arrangements in the AZ region during maturation. The red circle indicates the AZ location. Labels are in the lower right corner of each subpanel, and global annotation is positioned below.

To investigate the FAZ dynamics during fruit development in *A. arguta*, two cultivars with distinct abscission traits, ‘KL’ and ‘JL’, were selected. Eight samplings from 45 to 88 DPA established comprehensive developmental profiles (Figure 1A). Longitudinal sections revealed AZ formation at the pedicel–fruit interface. SEM analysis (Figure 1B) confirmed AZ initiation during early floral development in both cultivars, though their signal responsiveness diverged at comparable stages. Spatially, the AZ in ‘JL’ was embedded deeper within the fruit tissue (12.80 mm) than in ‘KL’ (9.45 mm) (Figure 1B). Temporally, ‘KL’ exhibited extensive AZ cell rupture, dissolution, and blurred cellular boundaries by 72 DPA, whereas ‘JL’ retained relatively intact AZ morphology relative to ‘KL’ at this stage (Figure 1B). These spatiotemporal differences in AZ positioning and differentiation likely drive the contrasting abscission behaviors between cultivars.

**Figure 2:**
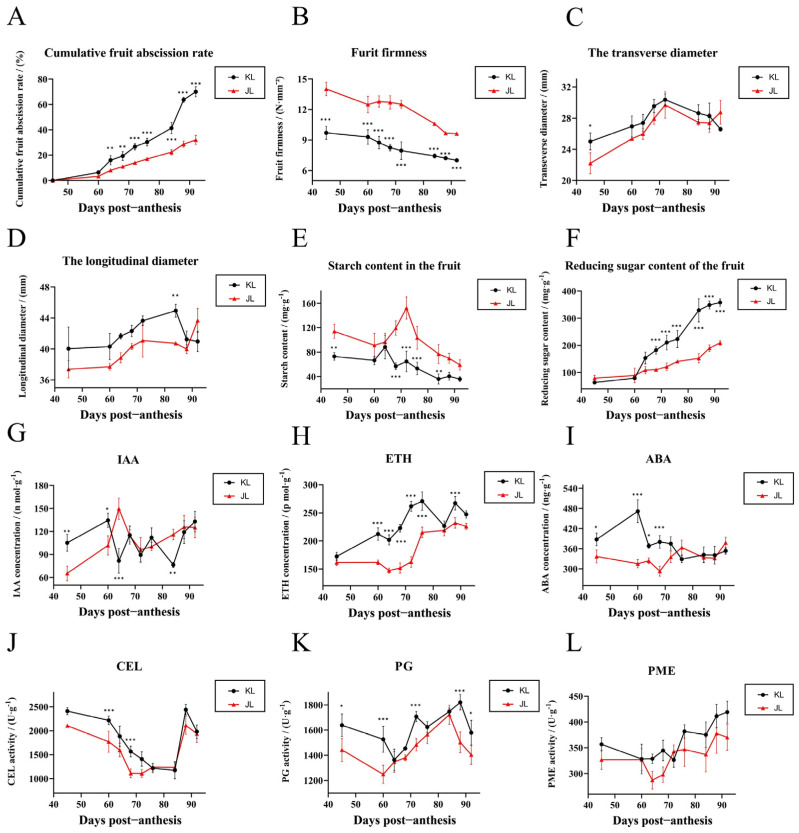
Physiological dynamics during fruit development in two *Actinidia arguta* cultivars. (**A**) Abscission rate showing two peaks in ‘KL’ (60, 84 DPA) corresponding to AZ cellular remodeling and full maturity, while ‘JL’ maintains low abscission. (**B**) Fruit hardness with cultivar-specific divergence, potentially linked to cell wall-modifying enzyme activities. (**C**,**D**) Fruit transverse and longitudinal diameters aligning with Figure 1A, indicating parallel developmental patterns. (**E**,**F**) Starch and reducing sugar contents exhibiting significant inter-cultivar differences. (**G**–**I**) AZ phytohormone levels (IAA, ETH, ABA) suggesting multihormonal regulation of abscission. (**J**–**L**) AZ enzymatic activities (CEL, PG, PME) reflecting differential cell wall degradation dynamics. Significant differences are indicated by asterisks (* *p* < 0.05, ** *p* < 0.01, *** *p* < 0.001).

Both cultivars exhibited parallel growth patterns in fruit transverse (Figure 2C) and longitudinal (Figure 2D) diameters, confirming their comparability as study materials for developmental analysis. Abscission data (Figure 2A) revealed two distinct peaks in ‘KL’: an initial peak at 60 DPA and a secondary peak at 84 DPA coinciding with physiological maturity markers (Figure 2B). Throughout development, ‘KL’ maintained lower fruit firmness and soluble starch content alongside elevated reducing sugar levels, suggesting metabolic coordination between these quality parameters.

### 3.2. Comparative Transcriptomic Analysis

#### 3.2.1. Transcriptomic Analysis and Enrichment Analyses of Differentially Expressed Genes (DEGs)

**Figure 3:**
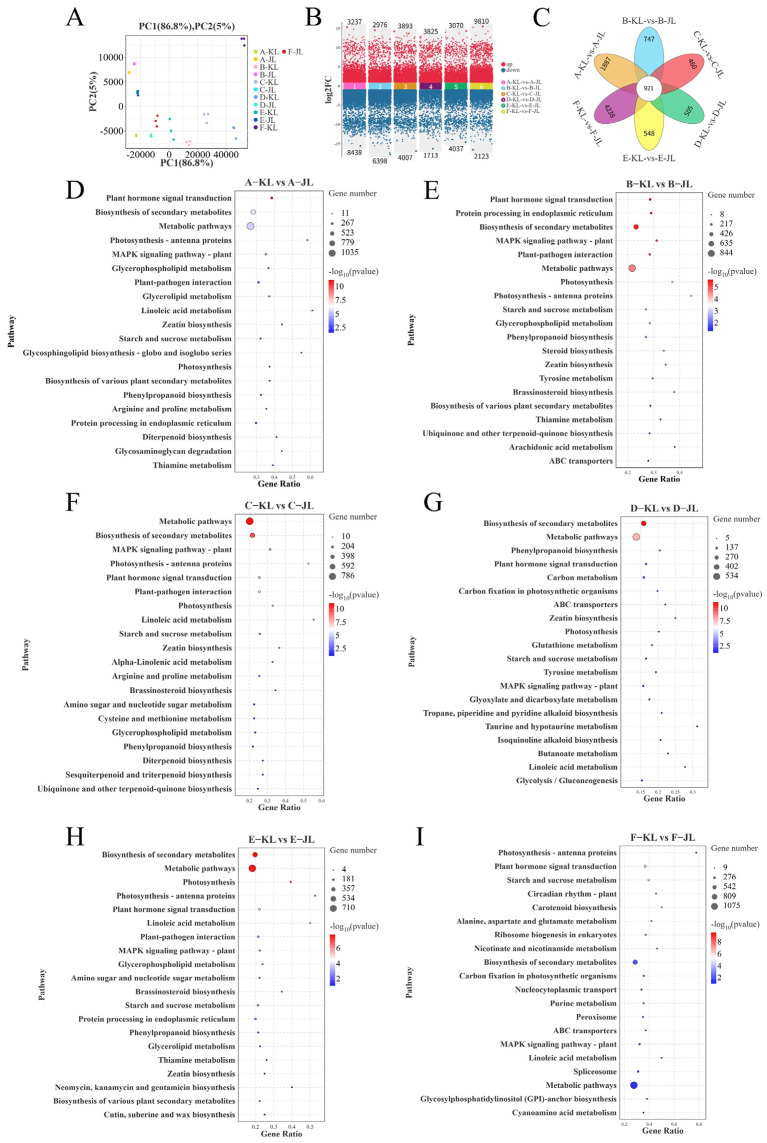
Transcriptomic analysis and enrichment analyses of DEGs. (**A**) Principal component analysis (PCA). (**B**) Different gene expression scatter plot. (**C**) Venn diagram of differences among six comparison groups. (**D**–**I**) KEGG pathway enrichment scatter plots of DEGs in different comparison groups ((**D**) A-KL vs. A-JL, (**E**) B-KL vs. B-JL, (**F**) C-KL vs. C-JL, (**G**) D-KL vs. D-JL, (**H**) E-KL vs. E-JL, (**I**) F-KL vs. F-JL).

To gain insights into the molecular mechanisms governing fruit abscission in *A. arguta*, two cultivars, ‘KL’ and ‘JL’, were selected, and six critical developmental stages (45 DPA, 64 DPA, 72 DPA, 76 DPA, 84 DPA, and 92 DPA), designated as stages A to F, respectively, were analyzed for transcriptomic sequencing. Quality control assessments revealed that the Q30 scores for all libraries exceeded 91.08%, and the GC content was over 46.00%, ensuring high-quality sequence assembly and subsequent analyses. PCA (Figure 3A) demonstrated excellent reproducibility within sample groups and significant differences between groups.

Six comparison groups were established by comparing the two cultivars at corresponding developmental stages. The criteria for identifying DEGs were set at false discovery rate (FDR) < 0.05 and fold change (FC) > 2. The statistical analysis (Figure 3B) identified the following numbers of DEGs for each stage from A to F: 11,675 (3237 upregulated/8438 downregulated), 9374 (2976 upregulated/6398 downregulated), 7900 (3893 upregulated/4007 downregulated), 5538 (3825 upregulated/1713 downregulated), 7107 (3070 upregulated/4037 downregulated), and 11,933 (9810 upregulated/2123 downregulated). Venn diagram analysis (Figure 3C) revealed 921 core DEGs shared among all six comparison groups. These genes exhibited highly conserved differential expression patterns during fruit abscission, suggesting they may reflect constitutive genetic divergence between cultivars while potentially harboring candidate genes regulating cultivar-specific abscission traits.

In order to gain deeper insights into the functional roles of DEGs associated with fruit abscission, we performed KEGG pathway enrichment analysis on the DEGs from the six comparison groups (Figure 3D–I). The results revealed significant enrichment in several key pathways across all comparison stages: plant hormone signal transduction (ko04075), biosynthesis of secondary metabolites (ko01110), metabolic pathways (ko01100), plant–pathogen interaction (ko04626), cytokinin biosynthesis (ko00908), starch and sucrose metabolism (ko00500), and photosynthesis (ko00195). These enriched pathways reflect the distinct physiological demands of the two cultivars during fruit development and maturation.

#### 3.2.2. Heatmaps of Gene Expression in Plant Hormone Signal Transduction and Cell Wall Synthesis/Degradation Pathways

**Figure 4:**
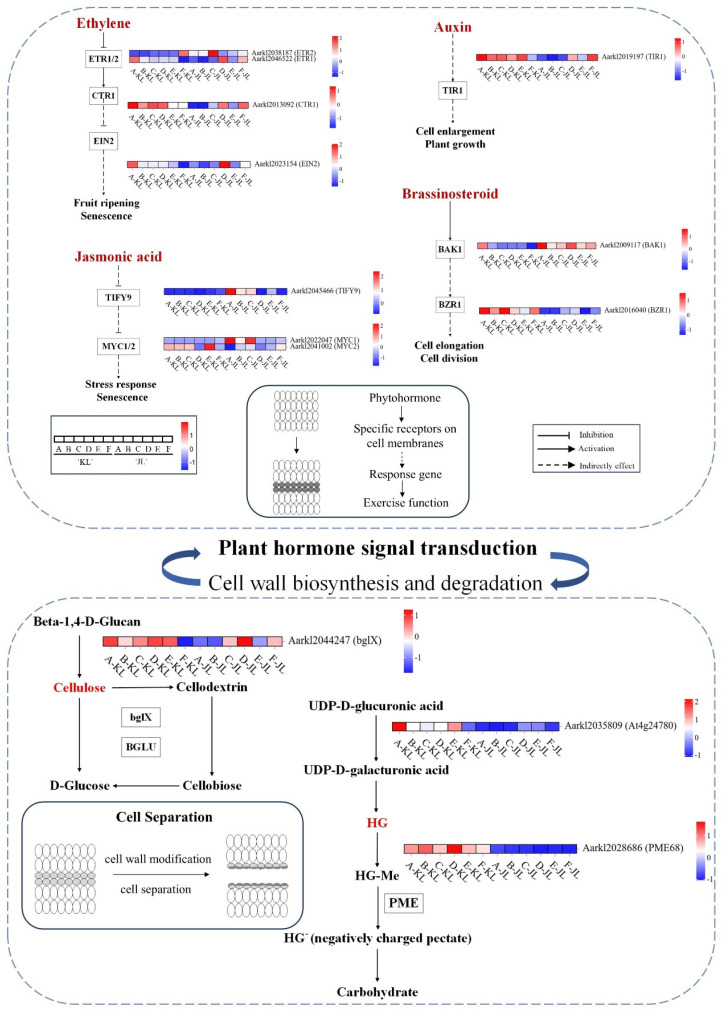
Schematic representation of key genes in hormone signaling and cell wall metabolism pathways. Heatmap of gene expression levels, from left to right: Stages A–F for ‘KL’ and Stages A–F for ‘JL’. Heatmap colors reflect normalized TPM values of genes across developmental stages. Solid/dotted lines denote direct/indirect regulatory interactions. Rounded rectangles outline simplified schematic representations of pathway components. Three candidate genes (*AabglX*, *AaAt4g24780*, *AaPAME68*) were prioritized based on transcriptional divergence. Partial genes with prominent expression differences are displayed; full heatmaps are provided in Supplementary Figure S4.

Based on transcriptomic data from two cultivars across six developmental stages, we generated heatmaps (Figure 4; full pathway maps in Supplementary Figure S4) to visualize plant hormone signaling pathways and biosynthesis/degradation routes of cell wall components (cellulose and pectin), elucidating their roles in fruit abscission in *A. arguta*. Heatmap analysis revealed that AUX responses in the FAZ were predominantly stage A-specific, whereas ETH, BR, JA, CTK, and ABA signaling pathways exhibited significant divergence throughout development, consistent with physiological assays (Figure 2). Specifically, cellulose biosynthesis genes (*AaCESA1*, *AaCESA2*, *AaCESA3*) and pectin synthesis enzymes (*AaGAE1*, *AaGAE6*) showed elevated expression in the early-to-mid stages in ‘JL’. Conversely, cellulose-degrading genes (*AaBGLU11*, *AaBGLU12*, *AaBGLU14*, *AaBGLU18*, *AaBGLU41*, *AaBACOVA02659*) and pectin-degrading enzymes (*AaPME1*, *AaPME7*, *AaPME18*, *AaPME31*, *AaPME34*, *AaPME59*, *AaPME68*) were upregulated in ‘KL’ during early-to-mid or all developmental stages, likely facilitating easier fruit abscission. These findings suggest that differential hormone signaling regulates cell wall-modifying enzyme activity, driving phenotypic divergence between cultivars.

**Figure 5:**
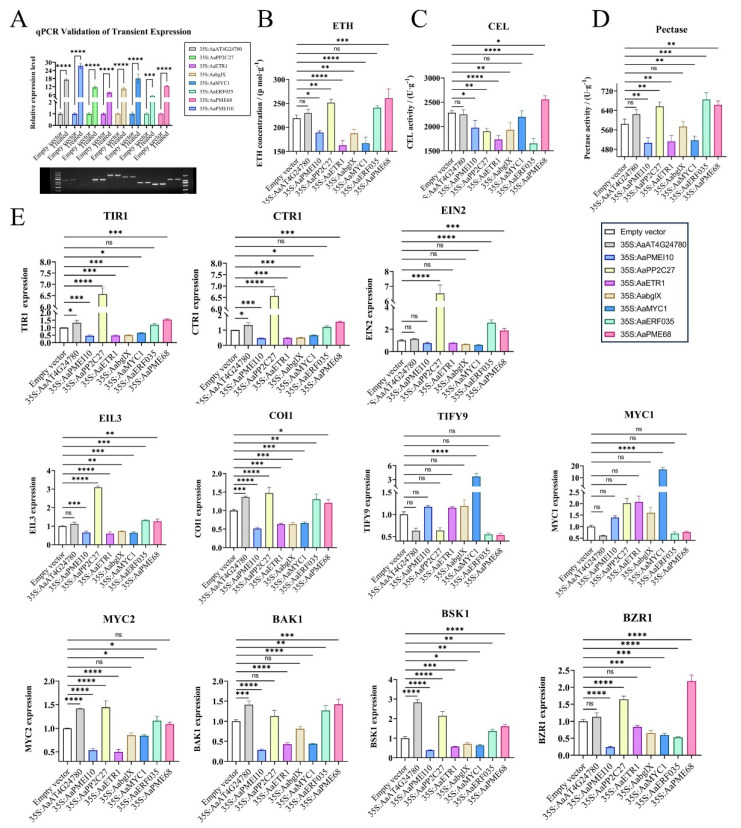
Transient transformation validation of candidate genes. (**A**) qPCR validation of transiently transformed candidate genes. Each candidate gene was overexpressed 5–25 times in the AZ of transiently transformed fruits. Gel electrophoresis results: from left to right, the bands are Trans5K DNA marker, *AaAt4g24780*, *AaPMEI10*, *AaPP2C27*, *AaETR1*, *AabglX*, *AaMYC1*, *AaERF035*, *AaPME68*, and Trans2K DNA marker. (**B**–**D**) ETH content and cell wall-modifying enzyme activities in the AZ of transiently transformed fruits ((**B**) ETH content, (**C**) CEL activity, (**D**) pectinase activity). (**E**) qPCR quantification of gene expression in the AZ of transiently transformed fruits. Significant differences are indicated by asterisks (* *p* < 0.05, ** *p* < 0.01, *** *p* < 0.001, **** *p* < 0.0001, ns *p* ≥ 0.05).

To further elucidate the critical role of ETH signaling in fruit abscission, we conducted overexpression studies of the ETH receptor *AaETR1* and the ETH response factor *AaERF035* in *A. arguta* fruits (Figure 5A). Our experiments revealed that overexpressing these genes not only influenced ETH production within the AZ but also modulated other plant hormone signaling pathways and cell wall-modifying enzyme activities (Figure 5B–E). In the AZ of *AaERF035*-overexpressing fruits, we observed significantly higher levels of ETH production and enhanced pectinase activity. The expression levels of key components in the ETH signaling pathway, such as *AaEIN2* and *AaEIL3*, were markedly upregulated, indicating robust activation of this pathway. In the AUX signaling pathway, the expression of AUX/IAA proteins (*AaIAA17*) was downregulated, leading to the release and activation of ARF, which subsequently activated downstream AUX-responsive genes. For the BR signaling pathway, the expression of BR receptor *AaBAK1* and BR response factor *AaBSK1* increased, enhancing BR-mediated responses. In the JA pathway, the overexpression of *AaERF035* led to elevated expression of the *AaMYC2* transcription factor, thereby activating JA signal transduction. In contrast, the overexpression of *AaETR1* resulted in excessive competitive binding to ETH, potentially inhibiting ETH signal transduction. This led to a significant reduction in cellulase and pectinase activities within the abscission zone and a decrease in the expression of the AUX receptor *AaTIR1*, thereby suppressing AUX signal transduction. In the JA pathway, the expression of *AaMYC1* increased while that of *AaMYC2* decreased, which partially inhibited JA signal transduction.

In order to investigate the key genes involved in the cell wall pectin degradation pathway, we conducted overexpression studies of *AaAt4g24780* (pectate lyase) and *AaPME68* (pectin methylesterase). The results showed that *AaAt4g24780* overexpression had no direct effect on ETH or cell wall hydrolases but indirectly influenced AUX signaling via AaTIR1. Conversely, the overexpression of *AaPME68* significantly elevated ETH levels and cellulase and pectinase activities within the AZ and influenced multiple plant hormone signaling pathways (ETH, AUX, BR). These results demonstrate that overexpressing *AaPME68* substantially accelerates fruit abscission in *A. arguta* by promoting pectin degradation through the hydrolysis of methyl esters in pectin.

#### 3.2.3. WGCNA Identifies Core Regulatory Genes Governing Abscission

**Figure 6:**
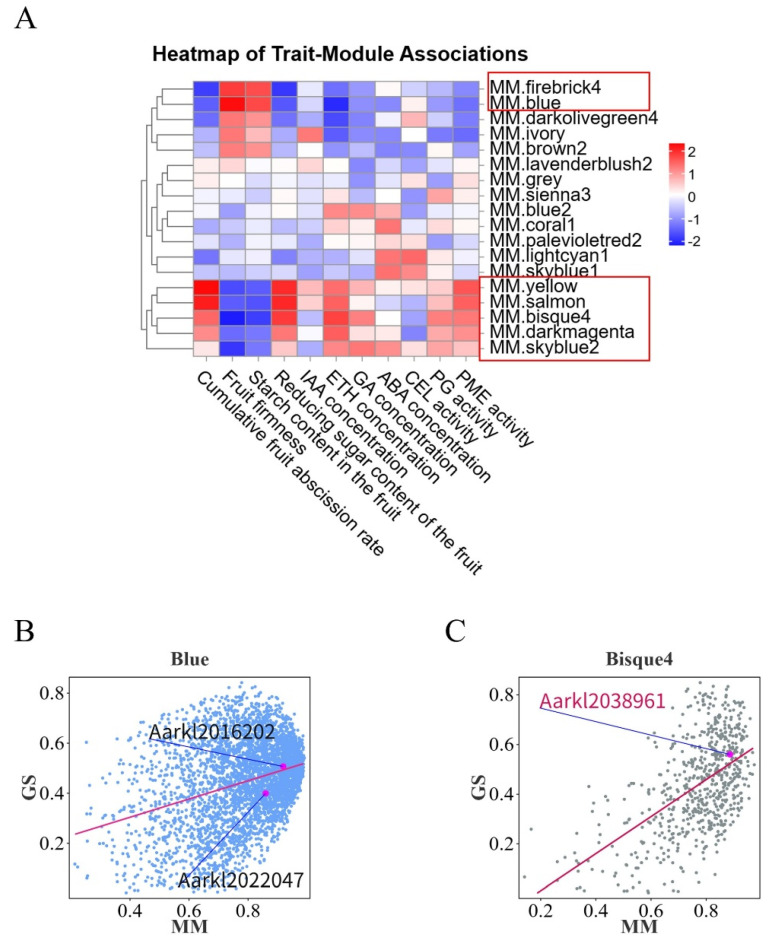
Weighted gene co-expression network analysis (WGCNA). (**A**) Trait–module association analysis. Seven modules significantly correlated with seven physiological indicators related to abscission were identified. The red-bordered frame denotes the screened candidate modules. (**B**,**C**) Membership–gene significance (MM-GS) analysis. Three highly correlated candidate genes were identified ((**B**) *Aarkl2016202*, *Aarkl2022047*, (**C**) *Aarkl2038961*).

To further identify key genes influencing fruit abscission in *A. arguta*, WGCNA was employed. This method clusters genes with similar expression patterns and correlates them with abscission-related traits to identify potential regulatory factors. In constructing the co-expression network, we selected β = 11 as the soft-thresholding power to ensure scale-free network topology (Supplementary Figure S7A). Using dynamic tree cutting and similarity calculations between module eigengenes, we assigned genes into 17 distinct color-coded modules and one gray module for unclassified genes (Supplementary Figure S7B). To determine which modules were most relevant to fruit abscission, we calculated Pearson correlation coefficients between each module’s eigengene (ME) and abscission traits, generating a trait–module association heatmap (Figure 6A). From this, we identified seven candidate modules with significant correlations. Further module membership–gene significance (MM-GS) analysis of these modules (Figure 6B,C) led to the identification of three core regulatory genes: *AaPP2C27* (*Aarkl2016202*), *AaMYC1* (*Aarkl2022047*), and *AaPMEI10* (*Aarkl2038961*). The functions of these genes in regulating fruit abscission were validated through transient transformation experiments (Figure 5A–E).

Protein Phosphatase 2C27 (*AaPP2C27*), functioning as a negative regulator in the ABA signaling pathway, significantly elevated ETH levels and pectinase activity while suppressing cellulase activity in the AZ upon overexpression. In the AUX signaling pathway, *AaTIR1* expression was markedly upregulated (Figure 5E). Within the ETH signaling pathway, the upregulated expression of *AaCTR1*, *AaEIN2*, and *AaEIN3* likely amplified ETH responses in the AZ (Figure 5E). In the JA pathway, *AaTIFY9* expression decreased, while *AaMYC1* and *AaMYC2* levels increased (Figure 5E). BR signaling was reinforced through significantly increased expression of *AaBAK1*, *AaBSK1*, and *AaBZR1* (Figure 5E). Collectively, *AaPP2C27* overexpression may promote ETH biosynthesis and modulate ETH, ABA, AUX, BR, and JA signaling pathways, accelerating fruit abscission via enhanced pectin degradation.

The basic helix–loop–helix (bHLH) DNA-binding superfamily protein *AaMYC1*, primarily suppressing JA signaling, significantly reduced ETH levels and pectinase activity in the AZ upon overexpression, with no notable effect on cellulase activity (Figure 5B–D). In the JA pathway, elevated *AaTIFY9* expression may indicate suppression of JA signaling, while BR signaling was attenuated through downregulated *AaBAK1*, *AaBSK1*, and *AaBZR1* (Figure 5E). These findings suggest that *AaMYC1* overexpression delays fruit abscission by potentially repressing JA signaling, reducing ETH production, and suppressing pectinase activity in the AZ.

Pectinesterase Inhibitor Domain Protein 10 (*AaPMEI10*), which delays pectin degradation by inhibiting pectin methylesterase activity, significantly suppressed cellulase and pectinase activities and markedly reduced ETH levels in the AZ when overexpressed (Figure 5B–D). In the AUX signaling pathway, *AaTIR1* expression was significantly downregulated (Figure 5E). ETH signaling components (*AaCTR1*, *AaEIN2*, *AaEIL3*) and BR-related genes (*AaBAK1*, *AaBSK1*, *AaBZR1*) were significantly downregulated (Figure 5E). Concurrently, *AaMYC2* expression declined (Figure 5E). These results imply that *AaPMEI10* overexpression may directly inhibit cell wall-modifying enzyme activities and attenuate fruit abscission by suppressing ETH biosynthesis and influencing AUX, ETH, BR, and JA signaling pathways.

### 3.3. Validation of Exogenous Plant Growth Regulator Treatments

**Figure 7:**
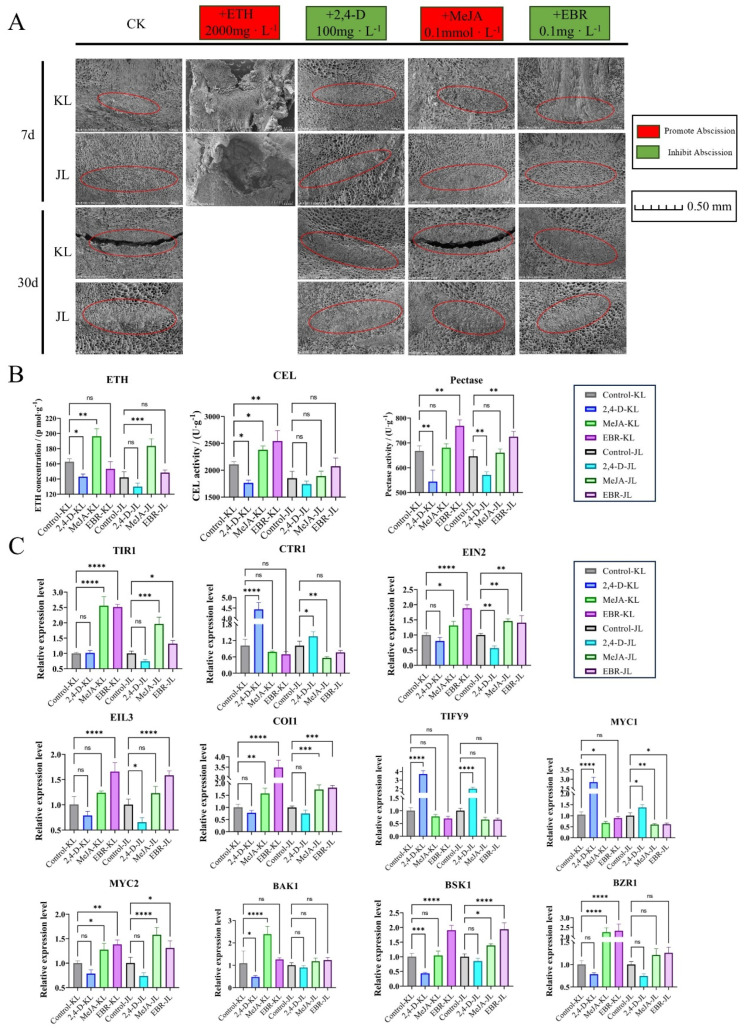
Validation of exogenous plant growth regulator treatments. (**A**): SEM images of AZ in *A. arguta* fruits of two cultivars after treatment with different growth regulators. The red circle indicates the AZ location. (**B**): ETH content and cell wall-modifying enzyme activities in AZ of two cultivars 30 days after treatment. (**C**): qPCR analysis of gene expression in AZ of two cultivars 30 days after treatment. Significant differences are indicated by asterisks (* *p* < 0.05, ** *p* < 0.01, *** *p* < 0.001, **** *p* < 0.0001, ns *p* ≥ 0.05).

This study systematically analyzed the developmental dynamics of FAZ cells in two *A. arguta* cultivars (‘KL’ and ‘JL’) under exogenous plant growth regulator treatments using SEM (Figure 7A). The water-treated control group provided a baseline for AZ cell development without hormonal intervention. Results revealed that in the control group, ‘KL’ exhibited distinct cellular rupture and cavity formation in AZ cells at 30 days post-treatment (DPT), whereas ‘JL’ failed to reach this developmental stage.

ETH treatment significantly accelerated softening and abscission in *A. arguta* fruit. By 5 DPT, both ‘KL’ and ‘JL’ displayed extensive premature fruit abscission accompanied by leaf senescence, with no residual fruits observed on branches by 7 DPT (Figure 7A). These findings indicate that ETH may play a decisive role in regulating fruit abscission in *A. arguta*.

Under 2,4-D treatment, AZ cells in both cultivars retained morphological similarity to the control group at 7 DPT, showing no premature development. Notably, even at 30 DPT, ‘JL’ AZ cells remained developmentally comparable to controls (Figure 7A), while ‘KL’ AZ cells lacked the cavity formation observed in controls, suggesting that 2,4-D may delay fruit maturation and AZ cell responsiveness to abscission signals. Quantitative analysis (Figure 7B) demonstrated that 2,4-D significantly suppressed ETH biosynthesis, CEL, and pectinase activities in ‘KL’ AZ. Gene expression profiling (Figure 7C) revealed upregulated *AaCTR1*, *AaTIFY*, and *AaMYC1* alongside downregulated *AaBAK1*, *AaBSK1*, and *AaBZR1*, implying that 2,4-D may regulate AZ activity by inhibiting ETH signaling and modulating JA/BR pathways. In ‘JL’, 2,4-D reduced pectinase activity without affecting ETH levels or CEL activity, while *AaEIN2* expression was suppressed and *AaTIFY9*/*AaMYC1* were upregulated.

Methyl jasmonate (MeJA) treatment potentially enhanced AZ cell responsiveness to abscission signals, with ‘KL’ exhibiting control-like cavity formation at 30 DPT, whereas ‘JL’ displayed pronounced cellular deformation at the fruit–pedicel junction (Figure 7A). Gene expression analysis (Figure 7C) indicated that MeJA significantly upregulated *AaTIR1*, *AaBSK1*, and *AaBZR1* in ‘KL’, potentially enhancing AUX signal transduction and BR pathway activation. In ‘JL’, MeJA upregulated *AaTIR1* and *AaCTR1* while downregulating *AaEIN2* and upregulating *AaMYC2*, suggesting its role in modulating AUX signaling and suppressing ETH responses to influence AZ development.

Epibrassinolide (EBR) treatment induced partial dissolution and rupture in ‘KL’ AZ cells, albeit with less severity than controls (Figure 7A). Quantitative analysis (Figure 7B) showed significantly elevated CEL and pectinase activities in ‘KL’ AZ at 30 DPT, alongside increased pectinase activity in ‘JL’. qPCR data (Figure 7C) revealed EBR-induced upregulation of *AaTIR1*, *AaEIN2*, *AaEIL3*, *AaMYC2*, *AaBSK1*, and *AaBZR1* in ‘KL’, potentially indicating BR pathway activation, enhanced AUX signaling, and ETH-mediated coordination of AZ activity. In ‘JL’, upregulated *AaEIN2*, *AaEIL3*, and *AaBSK1* alongside suppressed AUX signaling suggested BR-mediated fruit development regulation coupled with amplified ETH responses. Collectively, EBR primarily inhibited abscission during early developmental stages, with diminishing effects at later phases. Cultivar-specific variations in gene expression and enzymatic activity further imply that EBR may not serve as a primary determinant of abscission in *A. arguta*.

## 4. Discussion

### 4.1. Changes in the AZ During A. arguta Development

Abscission, which occurs in the AZ of plants, is a ubiquitous physiological process that significantly influences both yield and fruit quality. In horticultural plants, AZ can form at four distinct locations [21,22]: (1) the junction between fruit or floral tissues and pedicel tissues [23], (2) the midpoint of the pedicel [24], (3) the junction between pedicel tissues and peduncle tissues [25], and (4) the boundary between branch and peduncle [26]. The pedicel of *A. arguta* fruits consists of rigid xylem tissue, with a tight junction between the peduncle tissues and the branch. Physical breakage of the pedicel due to fruit weight occurs only during late maturation stages; however, this breakage does not occur at AZ. Using SEM observations (Figure 1B), we confirmed that the AZ in *A. arguta* forms primarily at the junction between fruit tissues and pedicel tissues. In the ‘KL’ cultivar, premature formation and differentiation of the AZ enable the fruit to exhibit a propensity for abscission early in development. Additionally, the AZ in ‘KL’ exhibits higher activity of ETH and cell wall-modifying enzymes during mid-development compared to the ‘JL’. Consequently, ‘KL’ displays more frequent abscission characteristics.

### 4.2. Transcriptomic Profiling and Functional Enrichment of Abscission-Associated Differentially Expressed Genes

The development of the AZ initiates during early floral development. When cells within the AZ begin to differentiate, the plant acquires the potential for abscission [27]; however, the capacity of AZ to respond to abscission signals is coordinately regulated by multiple factors. Through comparative transcriptomic analysis spanning six critical stages from early fruit development to full maturity, this study identified predominant regulatory factors underlying differential abscission characteristics between two *A. arguta* cultivars. Our analysis (Figure 3D–I) revealed significant enrichment of differentially expressed genes in KEGG pathways associated with plant hormone signaling, starch/sucrose metabolism, plant–pathogen interactions, and photosynthetic regulation during abscission. These molecular signatures align with established mechanisms reported in perennial fruit crops [5,28].

#### 4.2.1. Plant Hormone Signal Transduction Plays a Pivotal Role in Regulating *A. arguta* Abscission Process

Abscission signals within the AZ are directly modulated by plant hormone signaling pathways. Specifically, auxin efflux carriers, including AEC (auxin efflux carrier), PIN (PIN-formed), and PIL (PIN-like) proteins, play a critical role in reducing AUX concentrations within the AZ [29]. This reduction in AUX levels increases the AZ sensitivity to ETH, triggering the onset of the abscission process [30]. The analysis of plant hormone signaling pathways (Figure 4) revealed that the majority of genes involved in the AUX signaling pathway, including LAX, TIR1, and SAUR proteins, were predominantly active during the early stages of fruit development in both cultivars. Monitoring AUX content throughout fruit development (Figure 2G) indicated that AUX levels in the AZ of ‘KL’ cultivar start to decrease at 60 DPA, while in ‘JL’ cultivar, this reduction occurred at 64 DPA. The earlier reduction in AUX content in cultivar ‘KL’ is likely responsible for the more active and responsive abscission behavior observed in its AZ. Significant differences in many genes associated were observed with the ETH pathway within the AZs of the two cultivars across all six developmental stages. These variations in gene expression are likely a key factor underlying the differences in abscission characteristics between the cultivars.

Overexpression studies of key ETH signaling components *AaETR1* (ETH receptor) and *AaERF035* (ETH response factor) demonstrated that their elevated expression may substantially influence ETH biosynthesis and cell wall-modifying enzyme activities within the AZ, potentially exerting decisive effects on abscission progression (Figure 5B–D). Furthermore, *AaETR1* and *AaERF035* overexpression might modulate signal transduction of other phytohormones in the AZ, suggesting broader regulatory functions in fruit abscission. Enhanced ethylene signaling in the AZ could synergistically activate JA and BR pathways, as evidenced by upregulated expression of BR receptor *AaBAK1*, BR response factor *AaBSK1*, and JA response factor *AaMYC2* (Figure 5E). Exogenous plant growth regulator applications corroborated these findings (Figure 6A–C): ETH treatment induced growth cessation within 7 days, followed by rapid fruit softening and complete abscission in *A. arguta*, confirming ETH’s pivotal role in maturation–abscission coordination. Conversely, 2,4-D (an AUX analog) treatment significantly delayed fruit maturation, suppressed AZ responsiveness to abscission signals, and reduced both ethylene production and cell wall-modifying enzyme activity in the AZ compared to controls, indicating AUX-mediated antagonism of ETH effects.

Previous research has demonstrated that JA and AUX have antagonistic effects on the abscission process in plants [31]. AUX inhibits the expression of *SlJAR1* through *SlHB15A*, preventing the accumulation of JA-Ile (jasmonoyl-isoleucine), which in turn suppresses abscission. Comparative transcriptomics revealed stage-specific differential expression of JA pathway components, particularly JA response factors (*AaTIR1*, TIFY family proteins, *AaMYC1/2*), suggesting dynamic regulatory roles during fruit development. Exogenous MeJA treatment has been demonstrated to effectively promote fruit abscission [32]. In this study, MeJA application significantly enhanced abscission in both *A. arguta* cultivars (Figure 7A). However, by 30 DPT, cell wall-modifying enzyme activities in the AZ showed no significant difference from controls (Figure 7B). Further qPCR analysis (Figure 7C) suggested that MeJA might facilitate abscission by modulating AUX signaling pathways and suppressing ETH signaling.

BR signaling components—BR receptor *AaBAK1* and response factors *AaBSK1*/*AaBZR1/2*—exhibited significant expression variations across six developmental stages (Supplementary Figure S4). Exogenous EBR application delayed fruit abscission in both cultivars initially, though this inhibitory effect diminished progressively (Figure 7A). By 30 DPT, EBR-treated AZ tissues displayed consistently lower ETH content alongside enhanced pectinase activity compared to controls (Figure 7B). qPCR data (Figure 7C) suggested that BR signaling might participate in abscission regulation through coordinated AUX-ETH pathway interactions.

In summary, phytohormone signaling orchestrates *A. arguta* fruit abscission via dynamic AUX-ETH-JA-BR crosstalk. Efflux carriers (AEC, PIN, PIL)-mediated AUX depletion in the AZ enhances ETH sensitivity, initiating abscission. Cultivar-specific AUX decline timelines correlate with divergent abscission behaviors. Potentiated ETH signaling through *AaETR1* and *AaERF035* overexpression may drive cell wall degradation enzyme expression while coordinating the crosstalk between JA and BR signaling pathways via influencing regulatory factors such as *AaBAK1*, *AaBSK1*, and *AaMYC2*. Exogenous ETH accelerates abscission, whereas 2,4-D suppresses ETH-mediated maturation and enzymatic activity, highlighting their antagonism. JA counteracts AUX by inhibiting JA-Ile accumulation, while BR modulates abscission through AUX-ETH crosstalk. Transcriptomic and functional analyses reveal stage-specific expression of hormone-related genes (LAX, TIR1, AUX/IAA, TIFY, *AaBZR1/2*) and elucidate an integrated multihormonal regulatory network fine-tuning AZ activation.

#### 4.2.2. Effects of Carbohydrates on the Abscission of *A. arguta* Fruits

Carbohydrates play a dual role in regulating abscission. They influence the process through their impact on nutritional status and also contribute to the formation of the AZ by participating as polysaccharides. The presence of carbohydrates in the form of complex polysaccharides is crucial for the structural development and function of the AZ [33]. Ringing branches can increase the supply of carbohydrates to the fruits, which can prevent fruit abscission in citrus [34]. Silencing the hexokinase *LcHXK2* increases fruit abscission in litchi [35]. The analysis of soluble starch and reducing sugar content in fruits of two *A. arguta* cultivars during growth (Figure 2E,F) revealed distinct differences between the cultivars. ‘JL’ cultivar fruits maintain higher levels of soluble starch, providing a sustained supply of substrates for carbohydrate metabolism throughout the later stages of fruit development. Conversely, ‘KL’ cultivar fruits contain higher levels of reducing sugars. The accumulation of these sugars may lead to feedback inhibition, resulting in a decline in carbohydrate metabolic activity during later developmental stages. This metabolic decline could contribute to increased susceptibility to fruit abscission in the ‘KL’ cultivar. Besides the carbohydrate pathway, our analysis revealed that DEGs were also significantly enriched in the photosynthesis pathway. In horticultural plants, photosynthesis and carbohydrate metabolism are intrinsically linked. Light exposure plays a crucial role in regulating the accumulation of carbohydrates within the plant, positioning it as an upstream factor in carbohydrate metabolism. Under low light conditions, the abscission of cotton boll is closely linked to significant accumulation of hydrogen peroxide (H_2_O_2_) and flavonoids within the AZ. Concurrently, carbohydrate metabolism in the AZ is inhibited [36]. The result underscores the importance of managing carbohydrate dynamics and optimizing environmental factors like light exposure to mitigate fruit abscission of *A. arguta*.

#### 4.2.3. Cell Wall-Modifying Enzymes Are the Executors of Fruit Abscission in *A. arguta*

The activity of cell wall-modifying enzymes directly initiates the cellular separation process within the AZ and serves as an indicator of the fruit’s predisposition to abscission in this region. Cellulases (β-1,4-endoglucanases, CELs) hydrolyze cellulose, the main constituent of plant cell walls [37]. Xyloglucan endotransglucosylase/hydrolases (XTHs) participate in the breakdown of hemicelluloses [38]. Polygalacturonases (PGs), pectin lyases (PLs), and pectin methylesterases (PMEs) are tasked with dissolving the pectin-rich middle lamella [39]. EXPANSIONs (EXPs) play a role in relaxing the cell walls within the AZ [40]. Our analysis of CEL, PG, and PME activities during fruit development in two cultivars of *A. arguta* (Figure 2J–L) revealed significant differences in PG activity at multiple developmental stages between the cultivars despite generally consistent trends in the activity changes of all three cell wall-modifying enzymes. Comparative transcriptomic enrichment analysis of DEGs (Figure 3D–I) demonstrated significant expression differences in CESAs, bglX, GAEs, PMEs, PMEIs, GAUT1, and PGs genes across several periods in both cultivars. Overexpression studies of bglX, PME, and PMEI (Figure 5A–E) further substantiated the pivotal role of cell wall-modifying enzymes in the abscission process within the AZ of *A. arguta* fruits. Concurrently, key response genes in the AUX, ETH, and JA signaling pathways showed significant alterations in activity within the AZ of overexpressing fruits.

## 5. Conclusions

This study systematically elucidates the molecular and physiological mechanisms underlying fruit abscission in *A. arguta* through integrated comparative transcriptomics, transient genetic transformation, and exogenous hormone treatments. The results demonstrate that the contrasting abscission behaviors of the ‘KL’ (abscission-prone) and ‘JL’ (abscission-resistant) cultivars are governed by spatiotemporal differences in AUX depletion, ETH biosynthesis, and cell wall remodeling dynamics. Specifically, in the AZ of the ‘KL’, the early rapid decline of AUX levels synergizes with persistent ETH signaling activation to form a synergistic cascade (Figure 2G,H). This mechanism accelerates pectin degradation through enhanced activities of PG and PME. In contrast, delayed AUX reduction and suppressed ETH responses in ‘JL’ correlate with enhanced cell wall integrity and reduced abscission.

Functional validation via transient overexpression of *AaERF035*, *AaPME68*, *AaPP2C27*, *AaMYC1*, and *AaPMEI10* (Figure 5A–E) revealed that these genes may act as central regulators of hormone crosstalk (AUX, ETH, JA, BR) and cell wall metabolism. For instance, *AaERF035* and *AaPME68* may promote abscission by enhancing ETH biosynthesis and pectin hydrolysis, while *AaPMEI10* and *AaMYC1* may improve abscission resistance by inhibiting cell wall-degrading enzymes. Exogenous hormone treatments (Figure 7A–C) further confirmed that ETH and MeJA accelerate abscission, whereas AUX analogs (2,4-D) and EBR may delay AZ activation through antagonistic modulation of hormone pathways. Subsequent experimental validation will be required to further substantiate these findings.

This work advances our understanding of hormonal–transcriptional networks controlling fruit abscission and proposes actionable strategies for yield improvement in *A. arguta*, such as optimizing hormone management to enhance fruit retention. Future studies should explore the regulatory roles of carbohydrate metabolism and environmental factors (e.g., light intensity) in abscission, while the generated dataset provides a valuable resource for identifying key genes influencing this process.

## Data Availability

The raw sequence data reported in this paper have been deposited in the Genome Sequence Archive [41] in the National Genomics Data Center, China National Center for Bioinformation/Beijing Institute of Genomics, Chinese Academy of Sciences (GSA: CRA021978), and are publicly accessible at https://ngdc.cncb.ac.cn/bioproject/browse/PRJCA034730 (accessed on 16 March 2025).

## Supplementary Materials

Supplementary data to this article can be found online at https://data.mendeley.com/drafts/bfz2b49dw9.
